# Dietary patterns, cardiometabolic risk factors, and the incidence of cardiovascular disease in severe obesity

**DOI:** 10.1002/oby.20920

**Published:** 2015-04-10

**Authors:** David J. Johns, Anna‐Karin Lindroos, Susan A. Jebb, Lars Sjöström, Lena M. S. Carlsson, Gina L. Ambrosini

**Affiliations:** ^1^ Diet and Obesity Research, Medical Research Council, Human Nutrition Research, Elsie Widdowson Laboratory Cambridge UK; ^2^ The National Food Administration Uppsala Sweden; ^3^ Department of Primary Care Health Sciences University of Oxford, Radcliffe Observatory Quarter Oxford UK; ^4^ Institute of Medicine, University of Gothenburg Sweden; ^5^ School of Population Health, The University of Western Australia Perth Western Australia Australia

## Abstract

**Objective:**

The longitudinal associations between a dietary pattern (DP) and cardiometabolic risk factors and cardiovascular disease (CVD) incidence were investigated in a cohort of adults with severe obesity.

**Methods:**

The analysis included 2,037 individuals with severe obesity (>34 and >38 kg/m^2^ for men and women, respectively) from the Swedish Obese Subjects study repeatedly followed up for 10 years. Reduced rank regression was used to identify a DP characterized by dietary energy density, saturated fat intake, and fiber density. Mixed models examined relationships between repeated measures of DP *z*‐scores and cardiometabolic risk factors. Cox proportional hazards models assessed relationships between DP scores and CVD incidence.

**Results:**

An energy‐dense, high‐saturated‐fat, and low‐fiber DP was derived. A one‐unit increase in the DP *z*‐score between follow‐ups was associated with an increase in weight [*β* (SE)] (1.71 ± 0.10 kg), waist circumference (1.49 ± 0.07 cm), BMI (0.60 ± 0.34 kg/m^2^), serum cholesterol (0.06 ± 0.01 mmol/l), and serum insulin (1.22 ± 0.17 mmol/l; all *P* < 0.0001), as well as in serum triglycerides (0.05 ± 0.02 mmol/l; *P* < 0.05), systolic blood pressure (1.05 ± 0.27 mmHg; *P* < 0.001), and diastolic blood pressure (0.55 ± 0.16 mmHg; *P* < 0.05). No significant association was observed between repeated measures of the DP *z*‐scores and CVD incidence (HR = 0.96; 95% CI = 0.83‐1.12).

**Conclusions:**

An energy‐dense, high‐saturated‐fat, and low‐fiber DP was longitudinally associated with increases in cardiometabolic risk factors in severe obesity but not with CVD incidence.

## Introduction

Severe obesity increases the risk of morbidity and mortality 1, 2 with the heaviest men and women having a relative risk of 2.68 (1.76‐4.08) and 1.89 (1.62‐2.21), respectively, when compared with those with a normal BMI 2. Although the rate of increase in obesity prevalence may be slowing in some countries, there have been significant increases in the prevalence of severe obesity in the United States and Sweden 3, 4. Few studies have investigated the importance of diet in cardiovascular disease (CVD) in the presence of severe obesity. Frequently, the prevalence of severe obesity in cohort studies is insufficient to power separate analysis of these individuals.

Understanding the role of diet and its association with disease outcomes is of great importance as diet is one of the few modifiable lifestyle factors that may be targeted to bring about reductions in disease‐risk profiles. There is considerable evidence for saturated fat and dietary fiber playing a role in the development of CVD. Saturated fatty acids are associated with increases in total blood cholesterol and low‐density lipoproteins concentrations 5. A review update by the Cochrane heart group in 2011 suggested that studies reducing saturated fat intake or modifying the type of dietary fat consumed reduced the risk of cardiovascular events by 14% (95% CI = 4‐23%) 6. The greatest effect was seen in longer studies (over 2 years in length) that modified the type of fat intake rather than reducing it 6. This was confirmed in a meta‐analysis of the randomized control trials that replaced saturated fat intake with polyunsaturated fat intake for more than 1 year 7. Dietary fiber has been shown to decrease insulin secretion and improve glucose control, lower serum cholesterol levels, and lower blood pressure 8. Diets high in total dietary fiber are associated with a decreased risk for CHD and CVD 9, 10, 11. Dietary fiber may also impact on the energy density of the diet because of both its satiating effect and low energy density 12. A high‐energy‐density diet may promote passive overconsumption and desensitize appetite‐control mechanisms 13, thereby increasing obesity risk and subsequently CVD risk 14. Saturated fat intake, fiber intake, and dietary energy density (DED) may therefore play an important role in the development of CVD. However, the significance of these factors in CVD among the severely obese is not known.

Studying diet as a multidimensional exposure is closer to a real‐world scenario as foods consist of many nutrients, and diet is a combination of foods and nutrients. Dietary pattern (DP) analysis aims to reduce information collected on the reported intake of a large number of food items/groups into a few summary variables representing patterns of food and drink consumption. Reduced rank regression (RRR), a DP method, further allows the input of hypothesized pathways when deriving a DP thus enabling investigation of several closely related diet‐disease pathways together.

This study aims to investigate the longitudinal relationships between an energy‐dense, high‐saturated‐fat, low‐fiber DP, cardiometabolic risk factors (i.e., weight, waist circumference, serum lipids, insulin, and blood glucose), and cardiovascular events over a 10‐year period.

## Methods

### Study population

Data were obtained from the Swedish Obese Subjects Study (SOS) study 15. In brief, the SOS study is a national, ongoing prospective, matched control, and interventional study for obesity. SOS participants were pooled from a registry cohort of eligible individuals. Subjects had to be aged between 37 and 60 years and have a BMI of 34 or more for men and 38 or more for women. The registry population consisted of 6,905 individuals who underwent a health examination [time (*t*) = *R*]. From this registry population, 2,010 elected to have surgery and 2,037 were contemporaneously matched to the control group using 18 matching variables 15. Control subjects received the customary nonsurgical treatment for obesity at their given center of registration. No attempt was made to standardize the conventional treatment, which ranged from sophisticated lifestyle intervention and behavior modification to no treatment whatsoever. Allocation into the study arm at baseline (*t* = 0) occurred between September 1, 1987 and January 31, 2001.

Dietary data provided by the registry population (*n* = 6,897) were used to derive the DP. Subsequent longitudinal analysis used follow‐up data from the SOS control group only (*n* = 2,037).

Written informed consent was obtained for all study participants. The SOS study protocol was approved by the Research Ethics Committee of University of Gothenburg and seven other Swedish regional ethics review boards. The SOS trial has been registered in the ClinicalTrials.gov registry (NCT01479452, http://clinicaltrials.gov/ct2/show/NCT01479452?term).

### Data collection

#### Clinical and biochemical assessments

Participants were examined at registration (*t* = *R*), baseline (*t* = 0), and then after 0.5, 1, 2, 3, 4, 6, 8, and 10 years. Education status was as follows: five different levels classified into “up to 16 years” and “beyond 16 years” for this study; gender and age were recorded at baseline. At each examination, systolic blood pressure (SBP) and diastolic blood pressure (DBP) were averaged from repeat measures taken using standard methods. Body weight and height were also measured at each examination. Health questionnaires completed at each examination included questions on physical activity (ranged from 1 to 4, where 1 denotes sedentary activity and 4 regular strenuous exercise); smoking (dichotomized, currently smoking or not currently smoking); and self‐reported information on previous myocardial infarction (MI) and stroke. Participants also recorded the names of all drugs being taken in the last year, which were coded according to the Anatomical Therapeutic Chemical Classification system 16. Blood samples were obtained the morning after a 10‐ to 12‐h fast at *t* = *R*, 0, 2, and 10 years and sent to the Central Laboratory of Sahlgrenska University Hospital (accredited according to European Norm 45001) in Gothenburg. Total cholesterol, HDL cholesterol, and insulin were determined from serum samples. Blood glucose was also determined.

#### CVD incidence

Objective data on hospital care were obtained through data linkage with official Swedish registries. Information on first incidence of acute MI (ICD‐9: 410; ICD‐10: I21 and I22) or stroke (ICD‐9: 431, 433, 434, and 436; ICD‐10: I61, 163, 164, and 165) were obtained from the Hospital Discharge Register, which documents all hospital visits. For the purposes of the current analysis, follow‐up ended on June 16, 2009.

#### Dietary intake

A dietary questionnaire was completed at *t* = R, *0*, and after 0.5, 1, 2, 3, 4, 6, 8, and 10 years. The SOS dietary questionnaire is a semiquantitative dietary questionnaire that was adapted for the study population, considering the problematic eating characteristics of obese individuals. In addition, asking about the frequency of consumption of food items per day or per week as appropriate, as it places an emphasis on meal portion size, separated for weekdays and weekend. It uses color photographs to aid respondents and help to simplify the reporting of portion size of three basic food groups (vegetables, potatoes/rice/pasta, and meat/fish; Ref. 17). Based on the reported frequencies and portion size information, an algorithm calculated average intake per day of energy, macronutrients, alcohol, fiber, and selected micronutrients, using Swedish food composition tables 18. The questionnaire has been validated against 4‐day food diaries in obese and nonobese subjects. The correlations between the questionnaire and food records for nutrient values ranged from 0.34 (monosaccharides and disaccharides) to 0.62 (saturated fat; Ref. 17). A second validation with 24‐h energy expenditure and reported nitrogen intake found that underreporting errors did not increase with an increasing degree of obesity 19.

Foods were grouped into 39 groupings according to usual culinary usage and within the constraints of the food groupings in the dietary questionnaire 20. DED (kcal/g) was calculated for food intake only, excluding beverages 21. The percentage energy from saturated fat was calculated by dividing daily energy from saturated fat by total daily energy intake then multiplying by 100. Fiber density (FD; g/kcal) was calculated by dividing total dietary fiber intake (g) by total energy intake (kcal).

### Statistical analysis

Identification of the DPs has been previously described 20. In brief, RRR was applied using data from all subjects who provided dietary data at registration (*n* = 6,897) in order to identify DPs that best represent the Swedish obese population. The RRR model included the 39 food group intakes (g/day) as predictors, and DED, dietary FD, and percentage energy from saturated fat as response variables. These were chosen as response variables because of consistent evidence for their role in the development of CVD and weight gain. RRR identifies DPs (or patterns in food intake) that attempt to explain the maximum variation in response variables. Each participant is scored for each DP, using a *z*‐score. The DP *z*‐scores after *t* = *R* were estimated and standardized around the mean *z*‐score and standard deviation at *t* = R using a similar approach proposed by Imamura et al. 22. The DP scores at *t* = 0 to *t* = 10 therefore represent adherence to the pattern derived at *t* = R. The RRR models were run using the PLS procedure in SAS v9.1 23.

The relationships between DP *z*‐scores (exposure), CVD risk factors (outcome), and CVD events (outcome) were examined using mixed models. The mixed models of best fit (based on Akaike information criterion) included a random effect for individual and an unstructured covariance.

DP *z*‐scores at *t* = *R*, 0, 0.5, 2, 3, 4, 6, 8, and 10 were analyzed in relation with CVD risk factors measured at *t* = *R*, *t* = 0, *t* = 2, and *t* = 10 (PROC Mixed in SAS v9.1). The DP *z*‐score was the continuous exposure variable, and time since registration (years) was the continuous “time” variable. Models were adjusted for gender, education level, and age at registration, and smoking status, physical activity, and, where appropriate, blood pressure, lipid reducing, and diabetes medications at each follow‐up. To visualize longitudinal changes, average risk factor trajectories were generated from the models for tertiles of the DP score at baseline.

DP *z*‐scores at *t* = *R*, 0, 0.5, 2, 3, 4, 6, 8, and 10 were analyzed in relation with CVD incidence using Cox proportional hazard analysis (using stcox in STATA v11.2; Ref. 24). Participants who had a MI or stroke prior to the beginning of follow‐up (i.e., preregistry) were censored at baseline and those without an event were censored at a follow‐up cutoff of June 16, 2009, or at time of death. Gender, age, BMI, and physical activity and the use of diabetes, blood pressure, and lipid‐lowering medications at each follow‐up were considered as confounders in the models.

## Results

There were few differences in dietary, anthropometric, and lifestyle characteristics between the 6,869 individuals used to derive the DP and the control group followed up from baseline (*n* = 2,037; Table 1; Ref. 20).

**Table 1:** Characteristics of the registry population (*t* = *R*; *n* = 6,869) used to derive the DP and characteristics of the population followed up for 10 years as part of the SOS study control group (*t* = 0; *n* = 2,037) (Adapted from Ref. 20)

	*t* = *R*	*t* = 0		*t* = *R*	*t* = 0	
	Male			Female		
Measure	*n* = 2,605	*n* = 590	*P*‐value	*n* = 4,264	*n* = 1,447	*P*‐value
**Age (years)**	47.5 ± 5.9	46.8 ± 5.8	0.01	47.1 ± 6.1	47.6 ± 6.2	0.006
**Smoking (%)**	24.1	23.7	0.78	23.3	18.8	0.0003
**Weight (kg)**	121.9 ± 16.7	126.8 ± 14.4	0.001	112.6 ± 14.0	112.9 ± 13.9	0.52
**Height (m)**	1.79 ± 0.07	1.80 ± 0.07	0.001	1.65 ± 0.06	1.65 ± 0.06	0.80
**BMI (kg/m^2^)**	38.2 ± 4.6	39.2 ± 4.1	0.001	41.5 ± 4.4	41.6 ± 4.2	0.45
**Total energy intake (kcal)**	3,246 ± 1,238	3,160 ± 1,146	0.12	2,762 ± 1,144	2,705 ± 1,121	0.11
**Saturated fat intake (% total energy)**	15.8 ± 3.0	16.0 ± 3.1	0.24	16.5 ± 3.1	16.5 ± 3.1	0.93
**Fiber density (g/1,000 kcal)**	8.0 ± 2.5	8.0 ± 3.0	0.42	9.1 ± 2.5	9.4 ± 2.6	0.0002
**Dietary energy density (kcal/g)**	1.83 ± 0.38	1.82 ± 0.37	0.88	1.73 ± 0.37	1.70 ± 0.37	0.002

Data are means ± SD.

The DPs have been described previously 20. In brief, the first of the three derived DPs explained the majority of the variance (54%) in the response variables. The remaining two patterns combined explained only 26% of the total variation in response variables, and these were not taken forward for further investigation.

**Figure 1:**
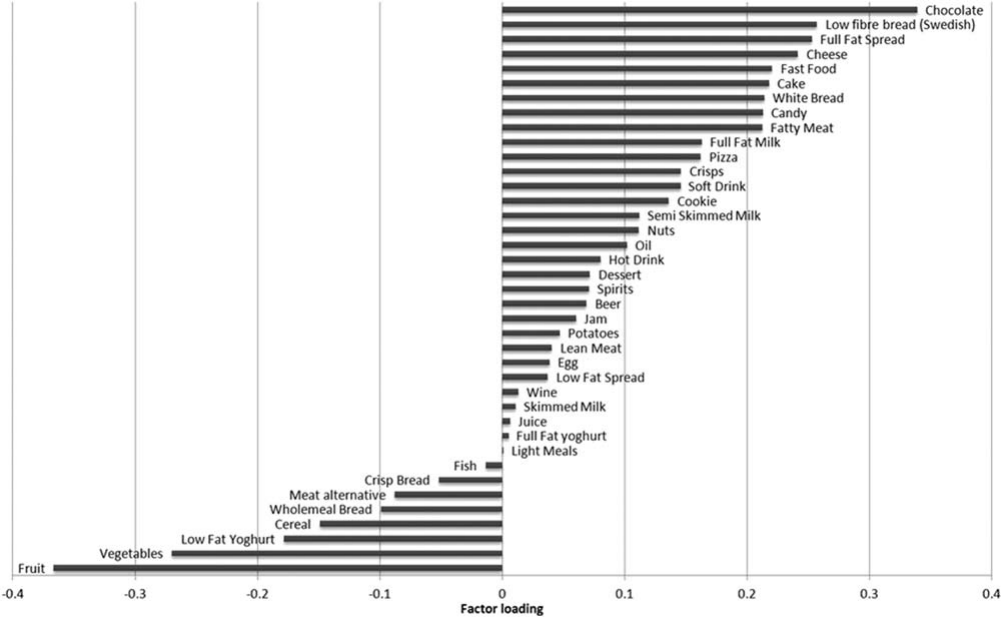
Factor loadings for the first DP high in energy density, high in percentage saturated fat, and low in fiber density 20.

The first DP was an “energy‐dense, high‐saturated‐fat, and low‐fiber DP” 20. A high DP *z*‐score was characterized by higher intake of chocolate, low‐fiber bread, cheese, fast food, and cake and low intake of fruit and vegetables (Figure 1; Ref. 20). The nutrient profile of the first DP has been published previously 20.

For the analyses of weight, waist circumference, blood pressure, and BMI as CVD risk factors, the number of observations ranged from 12,652 and 13,258. For blood glucose, serum insulin, cholesterol, HDL, and triglyceride outcomes, there were 4,166‐4,378 observations.

**Figure 2:**
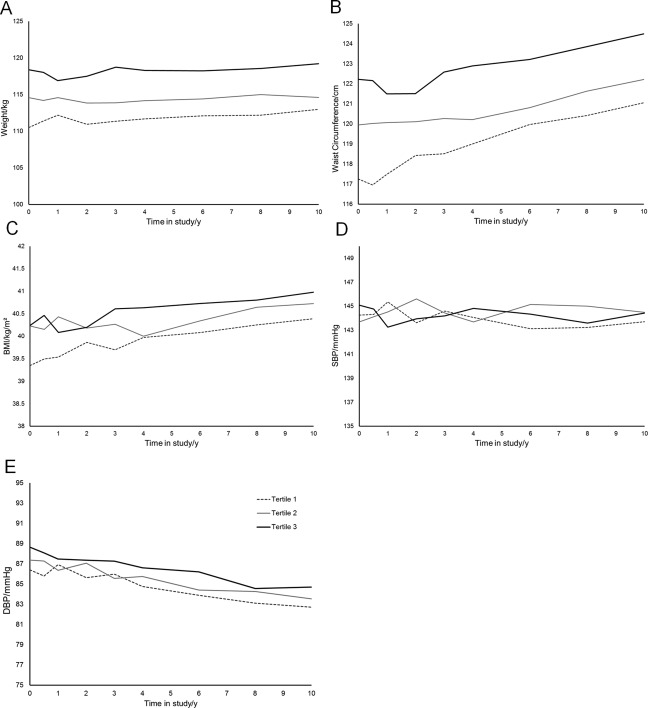
Average (**A**) weight, (**B**) waist circumference, (**C**) BMI, (**D**) systolic blood pressure (SBP), and (**E**) diastolic blood pressure (DBP) trajectories over 10 study years according to DP tertiles at baseline, as predicted from mixed model adjusting for sex, age, smoking, physical activity, and, where relevant, blood pressure‐lowering medication.

**Figure 3:**
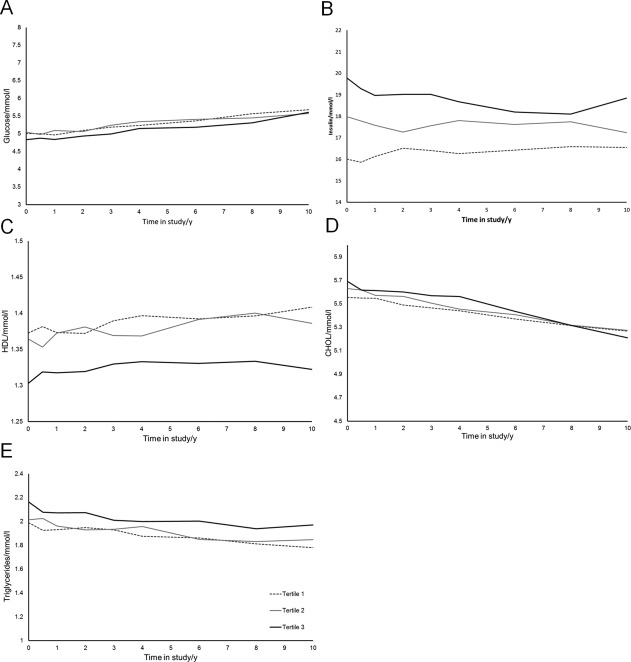
Average (**A**) blood glucose, (**B**) serum insulin, (**C**) HDL, (**D**) total cholesterol, and (**E**) triglyceride trajectories over 10 study years according to DP tertiles at baseline, as predicted from mixed model adjusting for sex, age, smoking, physical activity, and relevant medications.

Weight, waist circumference, BMI, and serum HDL all increased on average over time (Table 2; *P* < 0.0001). However, serum insulin, cholesterol, DBP, and serum triglyceride levels decreased on average over time (Table 2; *P* < 0.05). Blood glucose and SBP did not vary significantly with time. This can be observed in Figures 2 and 3, which show the average trajectories according to tertiles of the DP score at baseline, predicted by the longitudinal models.

**Table 2:** Longitudinal regression models showing the longitudinal association between repeated measures of the DP and CVD risk factors (DP *z*‐score) and the effect of time (i.e., aging) on these risk factors (time)

	DP *z*‐score		Time (years)	
	β (SE)	*P*‐value	β (SE)	*P*‐value
**Weight (kg)** a	1.71 (0.10)	<0.001	0.16 (0.03)	<0.001
**Waist circumference (cm)** a	1.49 (0.07)	<0.001	0.37 (0.03)	<0.001
**BMI (kg/m^2)^** a	0.60 (0.03)	<0.001	0.08 (0.01)	<0.001
**Blood glucose (mmol/l)** b	0.03 (0.02)	0.16	0.001 (0.006)	0.89
**Serum insulin (mmol/l)** c	1.22 (0.17)	<0.001	−0.10 (0.04)	0.026
**Serum Chol (mmol/l)** d	0.06 (0.01)	<0.001	−0.02 (0.003)	<0.001
**Serum HDL (mmol/l)** d	−0.001 (0.004)	0.86	0.003 (0.001)	<0.001
**Serum TG (mmol/l)** d	0.05 (0.02)	0.01	−0.02 (0.004)	<0.001
**SBP (mmHg)** e	1.05 (0.27)	<0.001	0.08 (0.08)	0.41
**DBP (mmHg)** e	0.55 (0.16)	0.03	−0.41 (0.05)	<0.001

a Adjusted for sex, age, smoking, and physical activity.

b Adjusted for sex, age, smoking, physical activity, serum insulin, and diabetic drug use.

c Adjusted for sex, age, smoking, physical activity, blood glucose, and diabetic drug use.

d Adjusted for sex, age, smoking, physical activity, and cholesterol‐lowering medication.

e Adjusted for sex, age, smoking, physical activity, and blood pressure‐lowering medication.

**Table 3:** The hazard ratio of CVD incidence for a one‐unit increase in the energy‐dense, high‐saturated‐fat, and low‐fiber DP using repeated measures of diet in 2,037 severely obese individuals

	Hazard ratio (95% CI),
	CVD incidence
**Unadjusted**	1.08 (0.95‐1.24)
**Model 1** a	1.09 (0.95‐1.26)
**Model 2** b	1.03 (0.90‐1.19)
**Model 3** c	0.98 (0.84‐1.14)
**Model 4** d	1.02 (0.83‐1.26)

a Adjusted for age and sex.

b Model 1: smoking.

c Model 2: physical activity and BMI.

d Model 3: lipid‐ and blood pressure‐modifying medications.

Between follow‐ups, a one‐unit increase in the DP score was longitudinally associated with, on average, a greater weight (1.71 ± 0.10 kg), waist circumference (1.49 ± 0.07 cm), BMI (0.60 ± 0.03 kg/m^2^), and serum insulin (1.22 ± 0.17 mmol/l; Table 2; all *P* < 0.0001). A one‐unit increase in the DP between follow‐ups was associated with, on average, greater serum cholesterol (0.06 ± 0.01 mmol/l; *P* < 0.0001), serum triglycerides (0.05 ± 0.02 mmol/l; *P* < 0.05), SBP (1.05 ± 0.27 mmHg; *P* < 0.001), and DBP (0.55 ± 0.16 mmHg; *P* < 0.05; Table 2). There was no significant association between the DP and blood glucose or serum HDL.

The analysis of CVD incidence was based on 29,904 person years of follow‐up and 211 (99 MI and 112 stroke) events. No significant association was observed between repeated measures of the energy‐dense, high‐saturated‐fat, and low‐fiber DP *z*‐scores and CVD incidence (HR = 0.96; 95% CI = 0.83, 1.12) after adjustment for age, sex, smoking, physical activity, and CVD relevant medication use (Table 3).

## Discussion

In this group of severely obese individuals, an energy‐dense, high‐saturated‐fat, and low‐fiber DP score was associated with significantly greater weight, waist circumference, BMI, serum insulin, cholesterol, triglycerides, SBP, and DBP during a 10‐year follow‐up. However, it was not associated with CVD incidence. These findings suggest that associations between the DP and CVD risk factors were present at baseline and follow‐up but that other competing factors influenced CVD incidence during the follow‐up of this severely obese adult cohort.

The associations observed in this obese group are similar to those in previous studies of DPs and CVD risk 25, 26, 27, 28, 29. They suggest an undesirable association between a DP characterized by high intake of snack foods and fast food and low intake of fruit and vegetables and cardiometabolic risk factors. Drogan et al. 30 identified a RRR DP in the EPIC‐Potsdam Study using nutrient densities of the dietary variables total fat, total carbohydrates, and fiber as response variables. The pattern, characterized by high intake of whole‐grain bread, fruits, fruit juices, grain flakes and/or cereals, and raw vegetables and a low intake of processed meat, butter, high‐fat cheese, margarine, and meat other than poultry, was similar to that found in the current study. In contrast to our findings, this DP was associated with a decreased risk of fatal but not nonfatal CVD events (RR across DP quartiles 1.00, 0.85, 0.31, and 0.47; *P* for trend = 0.016). It is, however, difficult to make clear comparisons with the current severely obese population as the EPIC‐Potsdam cohort used had a mean BMI of 27 kg/m^2^. It may be hypothesized that the greatest impact on CVD risk may already have occurred in these already severely obese individuals. This may be evident in the observation that only major changes in weight, brought about by bariatric surgery, have been shown to impact on CVD incidence after a 10‐year follow‐up in the severely obese 31. Furthermore, our DP was derived to explain the maximum variation in DED, saturated fat, and fiber; it is possible that another DP explaining other intakes could show different associations with CVD incidence.

There remains interest in the association between individual nutrients and/or foods and the risk of CVD; however, a shift in the thinking of nutritional epidemiologists has led to a number of studies considering the effects of overall DPs. DP analysis has frequently identified, in several cohorts, two DPs, often named the “Western” and “prudent” patterns. A Western DP is often characterized by higher intake of margarines and oil, sausages, coffee, butter, and full‐fat milk. The prudent pattern is often characterized by greater intake of vegetables, fruit, whole grains, fish, and poultry 32. The Western pattern has been positively associated with CVD incidence and cardiometabolic risk factors 32, 33; the converse is true for the prudent pattern 32, 33.

A number of clinical trials have also examined DPs. The DASH trial was based on a DP with high intake of fruits and vegetables and low‐fat dairy products and successfully reduced DBP and SBP and serum homocysteine levels in hypertensive subjects 34, 35. The PREDIMED study focused on the use of a Mediterranean DP and has shown that among people at high cardiovascular risk, a Mediterranean diet can reduce incidence of major cardiovascular events 36. However, to our knowledge, all of these observational and clinical trials have been conducted in populations with a lower mean BMI than this severely obese population.

The strength of this study was the use of repeated measures of diet and biological CVD risk factors. Furthermore, the SOS control group is the largest cohort of severely obese individuals in Europe and provides a unique opportunity to investigate diet in a previously understudied population. However, this group of individuals was required to meet a number of inclusion criteria reducing their heterogeneity, and despite choosing not to have surgery, these individuals volunteered to be part of the study and as such are likely more health conscious than the general severely obese population. We attempted to minimize this effect by deriving DP in a broader sample of severely obese individuals. Finally, the prevalence of dietary misreporting is often high in the obese population. The SOS dietary questionnaire was designed with this in mind and has been shown to be a valid measure of diet 17 with misreporting no greater than in the general population 18. However, misreporting does still exist and thus may influence results.

## Conclusion

In this severely obese population, measurements of an energy‐dense, high‐saturated‐fat, and low‐fiber DP were positively associated with fasting serum insulin, total cholesterol, weight, waist circumference, and BMI over a 10‐year period. Despite unfavorable CVD risk, diet was not associated with CVD incidence in this severely obese cohort. The impact of diet on health may have already occurred in these individuals who are severely obese at baseline.
